# Response to the letter to the editor “Electrical flash burns due to switchboard explosion”

**DOI:** 10.1111/iwj.14036

**Published:** 2022-11-26

**Authors:** Chao Lian, Xuan‐Fen Zhang, Xue‐Lei Li, Xiao‐Jun Liu

**Affiliations:** ^1^ Department of Plastic Surgery Lanzhou University Second Hospital Lanzhou Gansu People's Republic of China; ^2^ Department of Plastic and Aesthetic Surgery Affiliated Changzhi People's Hospital of Changzhi Medical College Changzhi Shanxi People's Republic of China; ^3^ Department of Plastic and Aesthetic Surgery Nanfang Hospital of Southern Medical University Guangzhou Guangdong People's Republic of China

*Dear Editor‐in‐Chief*

We thank the readers for their valuable and interesting feedback on our recent article “Electrical flash burns due to switchboard explosion”.

They point out that modified moist occlusive burn therapy (MMOBT) may be an alternative treatment for electrical flash burns. Although moist occlusive burn therapy (MOBT) and moist exposure burn therapy (MEBT) had been widely used in burn units, the optimal treatment remains controversial. In 2012, Mabrouk et al. found that moist occlusive dressing (Aquacel[®] Ag) significantly improves the management and healing rate of partial thickness facial burns with better long‐term outcome compared with moist open dressing (MEBO[®]). Scar quality was improved in the occlusive group. The frequency of dressing changes, pain and patient discomfort were also reduced in the occlusive group.^1^ Nevertheless, in 2016, Soltan Dallal et al. found that occlusive dressing was more susceptible to microbial contamination and infections than exposure dressing.^2^ Due to the limitations of both burn therapies, a novel therapeutic strategy is urgently needed. Based on the studies by Winter and Hinman in the 1960s, a new therapeutic concept, that sterile polyethylene film might be used as a type of moist occlusive dressing, was first proposed.^3^, ^4^ Based on this hypothesis, we first create a successful paradigm for the evolution of traditional burn therapy. To distinguish it from traditional MOBT, modified moist occlusive burn therapy (MMOBT) is first named by our team.^5^ MMOBT not only combines the advantages of both MOBT and MEBT but also eliminates the major disadvantages of both therapies. First, compared with MOBT, sterile polyethylene film effectively avoided the avulsion of new granulation tissue and alleviated the suffering of patients during dressing changes. Second, the transparent film allowed direct and close observation of the changes and healing of wounds. Third, compared with MEBT, the sterile polyethylene film covering could create a relatively closed and moist environment that could accelerate the speed of epithelization.

However, according to the readers' perspective, there are still three shortcomings as follows.

First, the number of cases in our study was relatively small, which could reduce the credibility. Actually, due to a low morbidity of electrical flash burns, it is really hard to collect sufficient samples within a short time. Undoubtedly, we promise to incessantly collect as many cases as possible in future studies. It is hoped that we can achieve more substantive results and share more practical clinical experiences.

The second shortcoming is the lack of comparison of the safety and efficacy with other topical agents. In general, surgical debridement and covering with burn cream is the primary choice for emergency management of burn injuries. As shown in our previous study, MMOBT is conducted by the combination application of chitosan‐based biogel and a layer of sterile polyethylene film.^5^, ^6^ At a 1‐year posttreatment follow‐up visit, most of the patients had achieved an acceptable aesthetic resurfacing (Figure 1). To carry out an in‐depth research, other typical medicines involving Moist Exposed Burn Ointment (MEBO) have been recently utilised to MMOBT as a potential alternative agent for the treatment of cervicofacial burns including scald burns.^7^ According to our experience, despite the satisfactory outcome, MEBO can only serve as an alternative burn cream but not as the preferred choice. To our knowledge, MEBO is an oil‐based herbal paste with brown colour and sesame‐oil smell, purported to be effective in the treatment of burn injuries and commonly used in Asia.^8^ Thus, to achieve sufficient drainage and distinct observation in support of better therapeutic effect, we prefer to use a kind of burn cream with characteristics of colourless, odourless and non‐oily, just like chitin. At a 1‐year posttreatment follow‐up visit, the patient had achieved a favourable aesthetic restoration without significant complications such as obvious dyspigmentation or scar contracture deformity. It is probably indicated that the key to the success of MMOBT is most likely the sterile polyethylene film. Due to the superiority of MMOBT, we suspect that this novel therapy may be applied equally to most second‐degree burns or superficial abrasions. To verify this hypothesis, further studies will be conducted in future.

**FIGURE 1:**
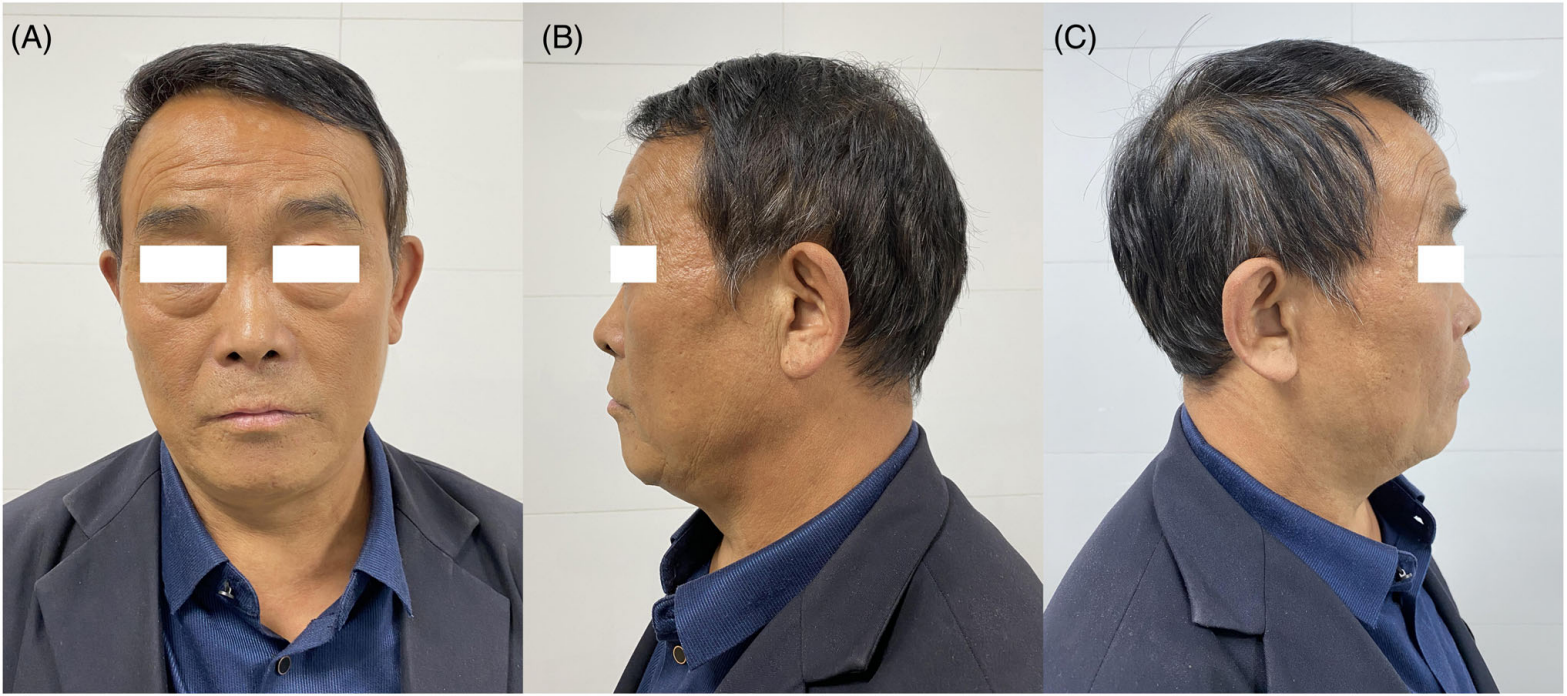
One‐year follow‐up result of electrical flash burns after MMOBT

Finally, the third shortcoming could be summarised as three points: (1) conceptual distinction between electrical injury and electrical flash burn; (2) difference between the consequences of high‐voltage current and low‐voltage current; (3) prerequisite condition for the occurrence of electrical flash burns. To illustrate the above issues, our reply is as follows:

1. Electrical injury may result from contact with faulty electrical appliances or machinery or contact with open household wiring or electrical power lines. The severity of electrical injury depends on the voltage, type of current and duration of contact with the source. High voltage always causes severe injuries. Higher than 1000 V is considered as a high voltage level, while lower than 1000 V is considered as a low voltage level.^9^
2. Low‐voltage injuries tend to cause small, deep contact burns at the exit and entry sites. However, high‐voltage injuries can be further divided into true high‐tension injuries and electrical flash injuries.^10^
3. True high‐tension burns are caused by high‐voltage current passing through the body, while electrical flash burns are caused by tangential exposure to a high‐voltage current arc where no current actually flows through the body (Figure 2). Obviously, the patient in our report consisted of the latter.

**FIGURE 2:**
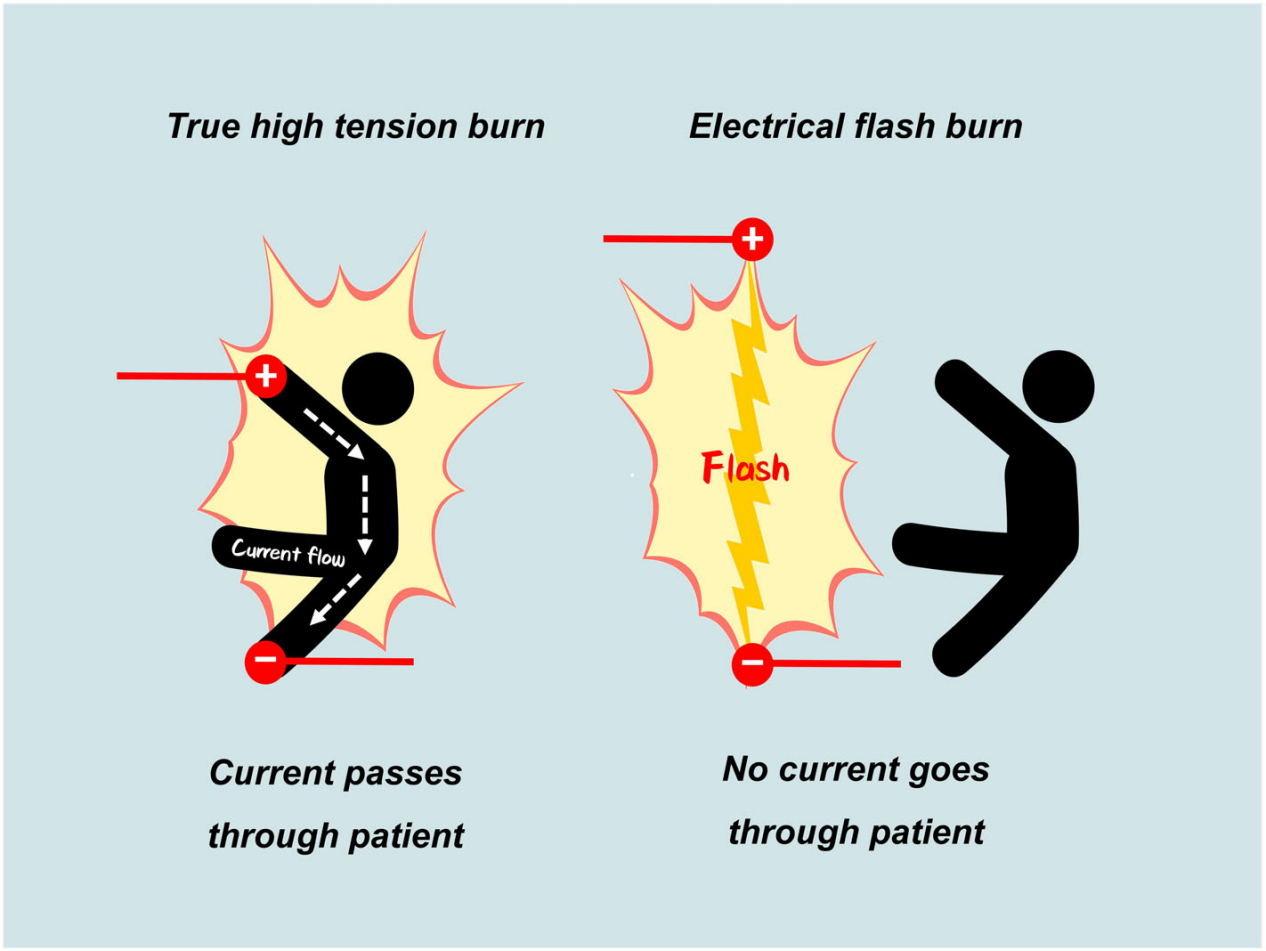
Distinction between true high‐tension burn and electrical flash burn

In summary, we concluded that MMOBT is a promising therapy for the treatment of cervicofacial burns. However, to confirm the superiority of MMOBT, further study is still needed.

## FUNDING INFORMATION

This study was supported by the Innovative Research Foundation of Changzhi People's Hospital (202005).

## CONFLICT OF INTEREST

The authors declare no conflicts of interest.

## Data Availability

Data sharing not applicable to this article as no datasets were generated or analysed during the current study.
